# Novel *OTOG* Variants and Clinical Features of Hearing Loss in a Large Japanese Cohort

**DOI:** 10.3390/genes16010060

**Published:** 2025-01-07

**Authors:** Yasuhiro Arai, Shin-ya Nishio, Shinichi Goto, Yumiko Kobayashi, Yohei Honkura, Akira Ganaha, Kotaro Ishikawa, Shin-ichiro Oka, Hiroshi Futagawa, Mayuri Okami, Fumio Takada, Kyoko Nagai, Tomoko Esaki, Takayuki Okano, Yumi Ohta, Shin Masuda, Kentaro Egusa, Masato Teraoka, Kazuma Sugahara, Shin-ichi Usami

**Affiliations:** 1 Department of Otorhinolaryngology-Head and Neck Surgery, Yokohama City University School of Medicine, Yokohama 236-0004, Japan; yasuarai@yokohama-cu.ac.jp; 2 Department of Hearing Implant Sciences, Shinshu University School of Medicine, Matsumoto 390-8621, Japan; nishio@shinshu-u.ac.jp; 3 Department of Otorhinolaryngology Head and Neck Surgery, Hirosaki University School of Medicine, Hirosaki 036-8562, Japan; goto-s@hirosaki-u.ac.jp; 4 Department of Otorhinolaryngology and Head and Neck Surgery, Iwate Medical University, Morioka 028-3694, Japan; ymhosoya0125@gmail.com; 5 Department of Otolaryngology-Head and Neck Surgery, Tohoku University School of Medicine, Sendai 980-8575, Japan; y-honkura@orl.med.tohoku.ac.jp; 6 Department of Otorhinolaryngology, International University of Health and Welfare, Narita Hospital, Narita 286-0124, Japan; ganaha.akira.t8@iuhw.ac.jp; 7 Department of Otolaryngology, National Rehabilitation Center for Persons with Disabilities, Tokorozawa 359-8555, Japan; ishikawa-kotaro@rehab.go.jp; 8 Department of Otorhinolaryngology, International University of Health and Welfare, Mita Hospital, Tokyo 108-8329, Japan; okashin@shinshu-u.ac.jp; 9 Department of Medical Genetics, Tokyo Metropolitan Children’s Medical Center, Tokyo 183-8561, Japan; 10 Department of Otorhinolaryngology, Tokai University School of Medicine, Isehara 259-1193, Japan; mayuri.okami@gmail.com; 11 Department of Medical Genetics and Genomics, Kitasato University Graduate School of Medical Sciences, Sagamihara 252-0373, Japan; ftakada@kitasato-u.ac.jp; 12 TAKASAKI Ear Nose and Throat Clinic, Takasaki 370-0031, Japan; hinakotamako11@yahoo.co.jp; 13 Department of Otolaryngology, Aichi Children’s Health and Medical Center, Obu 474-8710, Japan; tomotomo@rose.odn.ne.jp; 14 Department of Otolaryngology, Fujita Health University Bantane Hospital, Nagoya 454-8509, Japan; takayuki.okano@fujita-hu.ac.jp; 15 Department of Otorhinolaryngology-Head and Neck Surgery, Osaka University Graduate School of Medicine, Suita 565-0871, Japan; yota@ent.med.osaka-u.ac.jp; 16 Department of Pediatric Rehabilitation, Hiroshima Prefectural Hospital, Hiroshima 734-8530, Japan; s-masuda@hph.pref.hiroshima.jp; 17 Department of Otorhinolaryngology, Hiroshima City Hiroshima Citizens Hospital, Hiroshima 730-8518, Japan; kentaroegusa@yahoo.co.jp; 18 Department of Otolaryngology, Head and Neck Surgery, Ehime University Graduate School of Medicine, Toon 791-0295, Japan; mteraoka@m.ehime-u.ac.jp; 19 Department of Otolaryngology, Yamaguchi University Graduate School of Medicine, Ube 755-8505, Japan; kazuma@yamaguchi-u.ac.jp

**Keywords:** *OTOG*, otogelin, non-syndromic hearing loss, DFNB18B, congenital hearing loss, mild-to moderate hearing loss, non-progressive hearing loss

## Abstract

Background/Objectives: The *OTOG* gene is responsible for autosomal recessive non-syndromic sensorineural hearing loss and is assigned as DFNB18B. To date, 44 causative *OTOG* variants have been reported to cause non-syndromic hearing loss. However, the detailed clinical features for *OTOG*-associated hearing loss remain unclear. Methods: In this study, we analyzed 7065 patients with non-syndromic hearing loss (mean age 26.4 ± 22.9 years, 2988 male, 3855 female, and 222 without gender information) using massively parallel DNA sequencing for 158 target deafness genes. We identified the patients with biallelic *OTOG* variants and summarized the clinical characteristics. Results: Among the 7065 patients, we identified 14 possibly disease-causing *OTOG* variants in 26 probands, with 13 of the 14 variants regarded as novel. Patients with *OTOG*-associated hearing loss mostly showed congenital or childhood-onset hearing loss. They were considered to show non-progressive, mild-to-moderate hearing loss. There were no symptoms that accompanied the hearing loss in *OTOG*-associated hearing loss patients. Conclusions: We confirmed non-progressive, mild-to-moderate hearing loss as the clinical characteristics of *OTOG*-associated hearing loss. These findings will contribute to a better understanding of the clinical features of *OTOG*-associated HL and will be useful in clinical practice.

## 1. Introduction

Congenital hearing loss (HL) is among the most common sensorineural disorders, occurring in one of 500 to 1000 children. It is estimated that genetic factors contribute to more than half of congenital HL cases [1]. Over 120 genes are associated with non-syndromic HL, with approximately 20% of non-syndromic sensorineural HL being of autosomal dominant inheritance (the loci for autosomal dominant HL are denoted by DFNA with loci numbers) and approximately 60% being of autosomal recessive inheritance (the loci for autosomal recessive HL are denoted by DFNB with loci numbers). Autosomal recessive non-syndromic hearing loss (AR-NSHL) is a very heterogenous disorder, and 88 genes have been reported as genetic causes of ARNSHL (hereditary hearing loss homepage: https://hereditaryhearingloss.org (accessed on 19 January 2024)). Most cases of AR-NSHL are of prelingual onset and severe-to-profound HL. However, several *OTOG* gene variants are associated with AR-NSHL, presenting as mild-to-moderate sensorineural hearing loss [2].

The *OTOG* gene encodes the otogelin protein, a non-collagenous component of the acellular gelatinous matrices that envelop the sensory epithelia of the inner ear, the tectorial membrane (TM) in the cochlea, the otoconial membranes in the utricle and saccule, and the cupulae that cover the cristae ampullares of the semicircular canals in the vestibular organ [3]. Otogelin is also one of three major non-collagenous components in the tectorial membrane, together with α-tectorin and β-tectorin [4]. *OTOG* gene variants are known to cause AR-NSHL (DFNB18B, MIM 614945). To date, 44 causative variants in the *OTOG* gene have been reported (HGMD Professional: http://www.hgmd.org (accessed on 19 January 2024)). Moderate hearing impairment with a U-shaped to flat or slightly down-sloping-shaped audiogram is the most prevalent type of hearing impairment for *OTOG*-associated HL [4,5]. However, only a limited number of studies have revealed its detailed characteristics, including the onset age and long-term course of HL, and complications associated with vestibular symptoms have not been clarified.

In this study, we aimed to clarify the detailed clinical characteristics of *OTOG*-associated HL through a large number of HL patients carrying *OTOG* variants. We also present novel *OTOG* variants identified with massively parallel DNA sequencing (MPS) analysis.

## 2. Materials and Methods

### 2.1. Subjects

A total of 7065 Japanese HL patients (autosomal dominant or maternal inheritance, 1699; autosomal recessive or sporadic, 4542; inheritance unknown, 824) were registered, and genetic analysis was performed using MPS analysis for 158 target deafness genes. Among these subjects, we selected patients with biallelic *OTOG* variants. Written informed consent was obtained from all subjects (or guardians in the case of minors) prior to involvement in this study.

This study was approved by the Ethics Committee of the Shinshu University School of Medicine (no. 387—4 September 2012, no. 576—2 May 2017, and no. 718—7 March 2022) and other participating institutions listed previously [6]. This investigation was conducted in accordance with the Declaration of Helsinki and the approved study protocol.

Clinical characteristics (age, gender, medical history) and audiologic assessment data were obtained from medical charts. When audiograms were unavailable for infant cases, ASSR or conditional orientation audiometry (COR) threshold data were obtained. The hearing threshold (pure-tone average; PTA) was calculated by averaging the thresholds obtained at 0.5, 1, 2, and 4 KHz for the better hearing ear. The severity of HL was defined as follows: normal, <20 dB; mild impairment, 21–40 dB; moderate impairment, 41–70 dB; severe impairment, 71–90 dB; and profound impairment, >91 dB. The audiometric configurations were classified into low-frequency HL, mid-frequency HL (U-shaped HL), high-frequency HL, flat-type HL, and deaf, as reported previously [7].

### 2.2. Genetic Analysis

In this study, we analyzed 158 genes previously reported to be related with either non-syndromic or syndromic HL. The detailed protocol for super multiplex PCR and DNA sequencing is described elsewhere [8]. In summary, amplicon libraries were prepared using the Ion AmpliSeq Custom Panel, with the Ion AmpliSeq Library Kit 2.0 and the Ion Xpress Barcode Adapter 1–96 Kit (ThermoFisher Scientific, Waltham, MA, USA) in accordance with the manufacturer’s instructions. After preparing the amplicon libraries, an identical volume of the libraries for 45 patients were pooled. Ion S5 system sequencing was performed with an Ion 540 chip following the manufacturer’s instructions. The sequencing data were mapped against the human genome sequence (build GRCh37/hg19) using the Torrent Mapping Alignment Program. Subsequently, a variant call was performed with the Torrent Variant Caller software (version 5.16) included in the Torrent Suit (ThermoFisher Scientific). Following the variant call, the impacts of the variants were assessed using the ANNOVAR program [9]. Among the identified variants, missense, nonsense, insertion/deletion, and splicing variants were selected. Variants were further selected as detailed in our previous report [10]. The remaining *OTOG* gene variants were validated by direct sequencing. Direct sequencing was used to perform segregation analysis for family members.

The American College of Medical Genetics (ACMG) standards and guidelines [11] with Clingen HL expert panel specifications [12] were used to evaluate the pathogenicity of the identified variants. For variants previously reported as “Pathogenic” or “Likely Pathogenic”, the same pathogenicity classification was applied in cases without any contradictory evidence. The variants classified as “Likely Pathogenic” or “Pathogenic” in the ACMG guidelines were regarded as causative variants. Furthermore, variants classified as “Uncertain Significance” were also considered candidate variants if all of the following criteria were satisfied: (1) no other potential variants were detected in the other 157 genes, (2) the allele frequency was exceedingly low in the control populations of gnomAD, ToMMo 54KJPN, and in-house controls, (3) most of the in silico prediction scores support the pathogenic impact, and (4) no contradictory evidence exists regarding the pathogenicity of the identified variant.

### 2.3. Haplotype Analysis

The haplotype pattern across the 2 Mbp region surrounding the position of the frequent Japanese variation *OTOG*: NM_001277269: c.330C>G identified in this study was analyzed using a set of 29 single nucleotide polymorphisms (SNPs). We set this analysis region based on the recombination rates of the peripheral region calculated using the 1000-genome East Asian population. For this analysis, we selected seven patients with a homozygous c.330C>G variation. Sanger sequencing was employed to analyze haplotypes.

## 3. Results

### 3.1. Detected Variants

**Figure 1:**
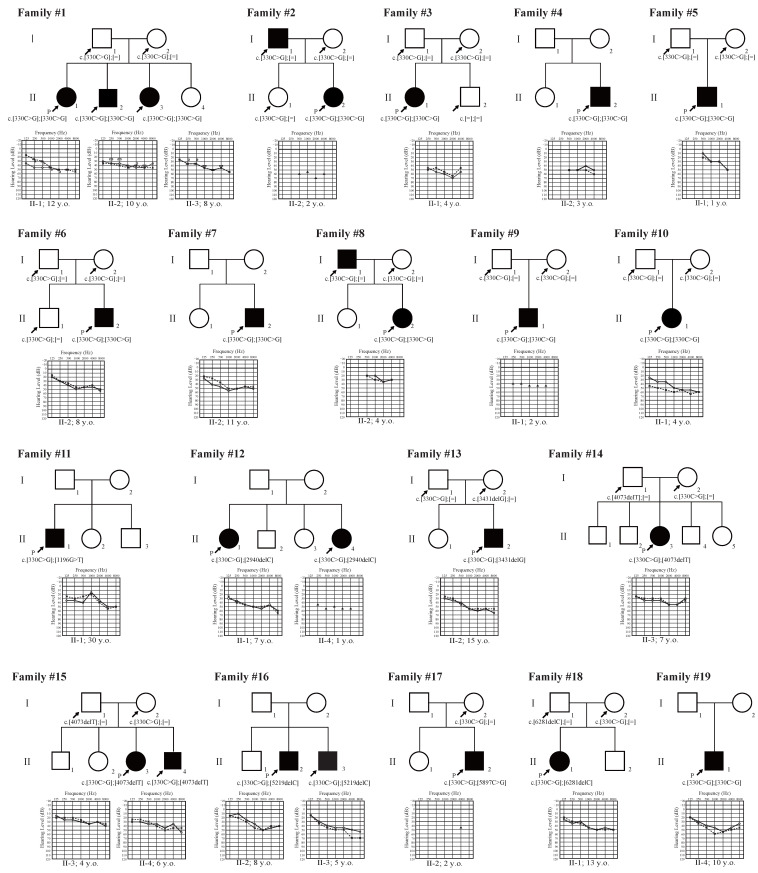

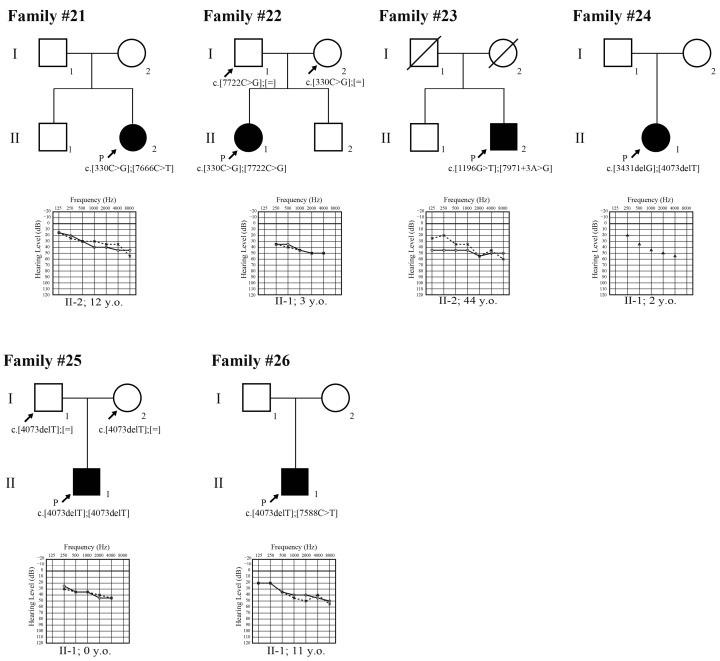
Pedigrees and hearing thresholds for the *OTOG*-associated HL patients identified in this study. Solid line: hearing threshold in the right ear; Dashed line: hearing threshold in the left ear.

We identified 14 possible disease-causing OTOG variants in 31 cases from 26 families, 13 of which were novel variants (Table 1). The novel variants consisted of three missense variants, four nonsense variants, one splicing variant, and five frameshift deletion variants. The minor allele frequency in the normal control database (ToMMo 54KJPN or gnomAD) for 12 novel variants was less than 0.0007, fulfilling the pathogenicity-supporting criterion in the Clingen HL expert panel specifications, but the minor allele frequency for c.4073delT exceeded this criterion slightly (MAF = 0.00076). Based on the ACMG guidelines, eight of the novel variants were categorized as “Likely Pathogenic” and five were categorized as variants of “Uncertain Significance”. Segregation analysis was performed with family member samples, with the results shown in Figure 1. In some cases, we could not complete segregation analysis, as we were unable to obtain peripheral blood samples from the family members.

### 3.2. Clinical Features of Patients with OTOG Variants

Among the 31 cases, no obvious syndromic symptoms other than HL were observed. Patient family history, audiograms, and clinical findings are summarized in Figure 1 and Table 2. The onset age or awareness age of the HL varied from 0 to 30 years old (mean age: 4.3 years). Most of the cases were first-decade-onset HL, and 15 of them had congenital HL. Only three individuals reported their HL after their first decade of life. We obtained hearing threshold data from 29 individuals from 26 families (Figure 1). All individuals exhibited bilateral and symmetrical HL. All cases for whom audiometric data were available had mild-to-moderate HL, including 10 cases with mild HL (34.5%) and 19 cases with moderate HL (65.5%). No individuals presented with severe-to-profound HL. The audiometric configurations were classified into flat-type HL in 23 cases (79.3%) and gently sloping high-frequency HL in six cases (20.7%). Among 26 cases, anamnestic assessment indicated that seven cases (26.9%) showed HL progression at the time of their genetic testing.

**Figure 2:**
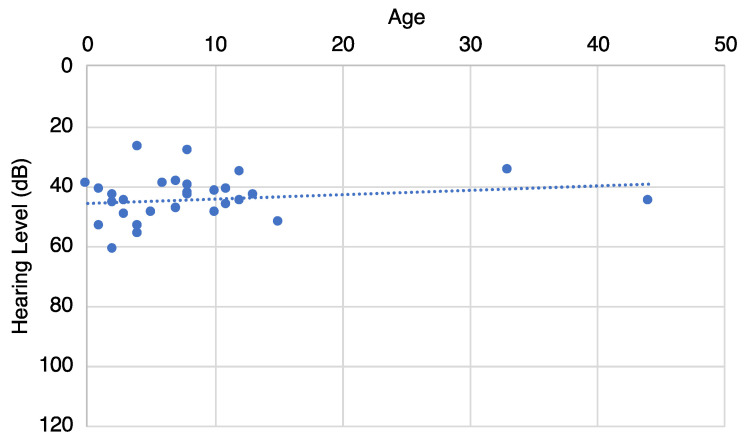
Detailed progression analysis of HL deterioration for patients with *OTOG*-associated HL. The dotted line indicates the linear regression. Each dot indicates the pure-tone average (PTA) and age of each patient. COR data were used instead of pure-tone audiometry in cases under the age of 5 y.o.

The pure-tone averages of the patients with *OTOG*-associated hearing loss and their ages at genetic testing are plotted in Figure 2. This figure reveals that the hearing levels were relatively stable and did not deteriorate with age.

Among the 26 cases with *OTOG*-associated HL for whom we obtained information regarding episodes of tinnitus and vestibular symptoms, we found that vertigo/dizziness was rare, and only one individual (JHLB-0441) had episodes of vertigo at the age of 22. Her right ear was found to have acute low-tone sensorineural hearing loss in addition to an episode of vertigo. Caloric testing, which represents the function of the semicircular canals, showed a decrease in response in her right ear. Furthermore, two cases complained of tinnitus.

### 3.3. Recurrent Variants

In this study, we identified a recurrent variant, c.330C>G, that was identified homozygously in 12 individuals and compound heterozygously with another variant in 14 cases. This variant has only been exclusively in Korean and Japanese HL patients [5,13]. To determine whether this variant originated from a common ancestor phenomenon or mutational hotspot, we performed a haplotype analysis using 29 single nucleotide polymorphisms (SNPs) (14 sites upstream and 15 sites downstream) surrounding the OTOG gene for seven patients from five families with the homozygous c.330C>G variant (Table 3).

To select the SNPs for haplotype analysis, Tag SNPs were selected using the SNPinfo web server (https://snpinfo.niehs.nih.gov (accessed on 16 December 2022)). SNP analysis was performed using direct Sanger sequencing. As a result of haplotype analysis, all patients were found to carry the same homozygous haplotype peripheral to the c.330C>G variant. This might be the result of linkage disequilibrium. The linkage disequilibrium range was 92173 bp for patients with c.330C>G mutations. Thus, we concluded that this mutation occurred due to founder effects, and diversification occurred via homologous recombination. This hypothesis was also supported by the fact that this variant is only observed in the East Asian population in the gnomAD database.

## 4. Discussion

In this study, we identified 31 individuals from 26 families with *OTOG* variants, including 13 novel variants, by MPS analysis. Although a total of 44 *OTOG* variants have been reported from various countries to date, this study is the largest analysis of *OTOG*-associated HL yet reported (HGMD professional ver.2023.4. http://www.hgmd.org (accessed on 19 January 2024)). Among the 13 novel variants identified in this study, only three variants were non-truncating variants and 10 were truncating variants. Thus, the underlying disease-causing mechanism for *OTOG*-associated HL appears to be biallelic loss of function variants leading to the lack of production of the functional otogelin protein. The c.4073delT variant identified in this study has a minor allele frequency slightly higher than the threshold defined by the ClinGen Hearing Loss expert panel (MAF = 0.0007). However, this variant was a frameshift deletion variant estimated to cause a loss of function, and all cases with this variant showed mild-to-moderate HL, which is characteristic of *OTOG*-associated HL. Thus, we regarded this variant to be candidate causative for *OTOG*-associated HL.

The prevalence of *OTOG*-associated HL among Japanese HL patients was 0.368% (26/7065). Interestingly, most of the cases carried homozygous or compound heterozygous c.330C>G variants. The minor allele frequency for c.330C>G in the Japanese normal control database (ToMMo 54KJPN: https://jmorp.megabank.tohoku.ac.jp) was 0.0039. Based on this carrier frequency, HL patients with homozygous c.330C>G variants were estimated to represent 0.38% of all SNHL patients. The prevalence in our study cohort (0.368%), including other *OTOG* variants, was comparable to or just a little lower than the estimation based on the carrier frequency in normal controls. This discrepancy could be due to enrollment bias in this study, as the majority of *OTOG*-associated HL cases had mild-to-moderate HL, were primarily followed-up in small clinics, and did not receive genetic testing.

With regard to the clinical characteristics of *OTOG*-associated HL, 70.0% (21/30) of cases had prelingual-onset HL. Among previous reports, all six papers (14 cases) reported prelingual onset [5,13,14,15,16,17]. All *OTOG*-associated HL cases identified in this study showed mild-to-moderate HL. The typical audiometric configuration for *OTOG*-associated HL was flat-type or gently sloping high-frequency HL. Six studies with audiometric configurations have been reported, and the majority of cases also showed flat-type or gently sloping high-frequency HL [4,5,13,14,15,18]. Interestingly, most of the cases with variants in tectorial membrane component genes, including *TECTA* [19], *COL11A2* [20], and *OTOA* [21], showed mild-to-moderate HL. Similarly to *OTOG*-associated HL, the audiometric configuration for *COL11A2*-associated HL was gently sloping high-frequency HL [22]. The *TECTA* gene encodes an α-tectorin protein consisting of three domains [19] that show various types of HL. The NIDO and ZA domain variants cause gently sloping high-frequency HL. In contrast, variants in the ZP domain show mid-frequency HL [23]. The *OTOA* gene, which encodes otoacorin, frequently shows mid-frequency HL [24]. The *OTOG* gene encodes the otogelin protein located in the acellular gelatinous membranes of the cupula, the otoconical membrane, and the tectorial membrane, along within Claudius cells, Hensen cells, and outer hair cells [3]. The otogelin protein is one of the tectorial membrane components and is also located in the horizontal top connectors and plays a crucial role in outer hair cell stereocilia bundling and enhances cochlear amplification [25]. Thus, the type of HL is expected to be similar to that in cases with other tectorial membrane component gene variants. This information will be beneficial in providing more appropriate medical care. For example, as part of genetic counseling, we can inform the patient that their hearing loss will not progress and is a good indication for hearing aids. Furthermore, as their hearing will not deteriorate rapidly, frequent follow-up is not required.

In terms of hearing deterioration, the results for our cohort results are shown in Figure 2 and clearly indicate that the hearing levels did not deteriorate with age. This is in line with previous reports that HL does not progress for 10 to 20 years [4,5,10]. Therefore, we concluded that *OTOG*-associated HL is a non-progressive form of sensorineural HL. However, since long-term hearing data were unavailable in this study, it cannot be ruled out that environmental factors or other factors may affect hearing deterioration, so further prospective studies will be needed.

The otogelin protein is expressed in the vestibule, particularly in the acellular gelatinous structures that cover the otoconial membranes in the utricle and saccule and is also expressed in the cupulae that cover the cristae ampulla of the semicircular canals [3]. However, only one patient reported symptoms of vertigo and showed unilateral canal paresis, while 25 patients had no symptoms. As the patient’s vertigo appeared to be accompanied with acute low-tone hearing loss, the cause of her vertigo might be due to endolymphatic hydrops or another cause of vertigo. Some previous studies reported that patients demonstrated bilateral dysfunction in caloric and rotation chair testing [2,4]. On the other hand, Yu et al. reported that one patient did not have any vestibular dysfunction in caloric or rotation chair testing. From our study results and previous reports, vestibular dysfunction appears to be quite rare among cases of *OTOG*-associated HL. As the limitation of this study, we did not perform comprehensive vestibular assessment (caloric testing, cervical vestibular evoked myogenic potential, ocular vestibular evoked myogenic potential, and video head impulse testing). Thus, further studies will be needed to conclude the effect of *OTOG* gene variants on vestibular function.

In this study, we identified one recurrent variant, c.330C>G, which was detected in 84.6% (22/26) of unrelated Japanese HL families. This variant has only been reported from Japanese and Korean HL patients to date. Based on the ethnically biased distribution of this variant, we hypothesized that this variant was caused by a common ancestor (founder mutation). Our haplotype analysis of homozygous c.330C>G cases also supports this hypothesis, and the same haplotype was detected in the peripheral region of c.330C>G variants. These results suggest that this variant occurred in a common ancestor of the East Asian population and spread among the Korean and Japanese populations via the founder mutation phenomenon.

## 5. Conclusions

In conclusion, we demonstrated the detailed clinical features of *OTOG*-associated HL patients in the largest cohort of patients studied to date. Among the 14 causative candidate variants, 13 variants were novel, with 10 of them being truncating variants. In most cases, the onset of HL was congenital or childhood in nature. The typical audiometric configuration of *OTOG*-associated HL was flat-type or gently sloping high-frequency mild-to-moderate HL. The hearing level was considered to be stable and non-progressive. Although the otogelin protein was expressed in the vestibular end organ, vestibular symptoms are expected to be rare among patients with *OTOG*-associated HL.

## Figures and Tables

**Table 1:** OTOG variants identified in this study.

Nucleotide Change	AA Change	Exon	SIFT	PP2	MutTaster	MutAssessor	REVEL	CADD	ToMMo 38KJPN	Gnomad All	Pathogenicity	Reference
c.330C>G	p.Tyr110*	Exon 4	.	.	A	.	.	29.6	0.00388	0.00001	Pathogenic	[13]
c.1196G>T	p.Cys399Phe	Exon 10	D	.	D	H	0.95	32	0.00030	0.00004	VUS	This study
c.2940delC	p.Pro981Leufs*43	Exon 24	.	.	.	.	.	.	.	.	Likely_Pathogenic	This study
c.3431delG	p.Ala1145Leufs*17	Exon 27	.	.	.	.	.	.	0.00003	.	Likely_Pathogenic	This study
c.4073delT	p.Val1358Glyfs*81	Exon 32	.	.	.	.	.	.	0.00076	.	VUS	This study
c.5219delC	p.His1742Thrfs*127	Exon 35	.	.	.	.	.	.	0.00004	,	Likely_Pathogenic	This study
c.5897C>G	p.Ala1966Gly	Exon 35	D	D	N	M	0.067	22.8	0.00006	.	VUS	This study
c.6281delC	p.Val2095Cysfs*24	Exon 36	.	.	.	.	.	.	.	.	Likely_Pathogenic	This study
c.7021C>T	p.Gln2341*	Exon 41	.	.	A	.	.	51	.	.	Likely_Pathogenic	This study
c.7523A>G	p.Asn2508Ser	Exon 44	D	.	D	M	0.201	26.7	0.00053	0.00001	VUS	This study
c.7588C>T	p.Gln2530*	Exon 44	.	.	A	.	.	50	0.00009	.	Likely_Pathogenic	This study
c.7666C>T	p.Arg2556*	Exon 45	.	.	A	.	.	55	.	.	Likely_Pathogenic	This study
c.7722C>G	p.Tyr2574*	Exon 45	.	.	A	.	.	45	0.00040	.	Likely_Pathogenic	This study
c.7971+3A>G		Exon 48	.	.	.	.	.	.	.	.	VUS	This study

All variants are indicated on NM_001277269. AA: amino acid; PP2: PolyPhen2; MutTaster: Mutation Taster; MutAssessor: Mutation Assessor; *: stop codon.

**Table 2:** Clinical characteristics of the OTOG-associated hearing loss patients identified in this study.

Family Number	ID	Relationship	Base Change Allele 1	AA Change Allele 1	Base Change Allele 2	AA Change Allele 2	Hereditary	Awareness	Age	Gender	Severity of HL	Type of HL	Fluctuation	Progression	Tinnitus	Vertigo	Hearing Aids
1	JHLB-0441	Proband	c.330C>G	p.Tyr110*	c.330C>G	p.Tyr110*	AR	9	12	F	Moderate	HF gentle	Y	N	N	Y	Y
	JHLB-0442	Brother	c.330C>G	p.Tyr110*	c.330C>G	p.Tyr110*		10	10	M	Mild	Flat	N	N	N	N	N
	JHLB-0443	Sister	c.330C>G	p.Tyr110*	c.330C>G	p.Tyr110*		7	8	F	Moderate	HF gentle	N	N	N	N	N
2	JHLB-0580	Proband	c.330C>G	p.Tyr110*	c.330C>G	p.Tyr110*	AD	0	2	F	Moderate	Flat	NA	NA	N	N	NA
3	JHLB-1308	Proband	c.330C>G	p.Tyr110*	c.330C>G	p.Tyr110*	Sporadic	0	4	F	Moderate	Flat	Y	Y	N	N	Y
4	JHLB-4118	Proband	c.330C>G	p.Tyr110*	c.330C>G	p.Tyr110*	Sporadic	0	3	M	Moderate	Flat	NA	Y	NA	NA	NA
5	JHLB-5053	Proband	c.330C>G	p.Tyr110*	c.330C>G	p.Tyr110*	Sporadic	1	1	M	Mild	HF gentle	N	N	N	N	Y
6	JHLB-5086	Proband	c.330C>G	p.Tyr110*	c.330C>G	p.Tyr110*	Sporadic	5	8	M	Moderate	Flat	NA	NA	N	N	N
7	JHLB-5177	Proband	c.330C>G	p.Tyr110*	c.330C>G	p.Tyr110*	Sporadic	0	11	M	Moderate	Flat	N	N	N	N	Y
8	JHLB-7360	Proband	c.330C>G	p.Tyr110*	c.330C>G	p.Tyr110*	Sporadic	0	4	F	Mild	Flat	N	N	N	N	Y
9	JHLB-7826	Proband	c.330C>G	p.Tyr110*	c.330C>G	p.Tyr110*	Sporadic	0	2	M	Moderate	Flat	N	N	NA	NA	Y
10	JHLB-8343	Proband	c.330C>G	p.Tyr110*	c.330C>G	p.Tyr110*	Sporadic	0	4	F	Moderate	Flat	N	N	N	N	Y
11	JHLB-5121	Proband	c.330C>G	p.Tyr110*	c.1196G>T	p.Cys399Phe	Sporadic	20	30	M	Mild	HF gentle	Y	Y	Y	N	NA
12	JHLB-10206	Proband	c.330C>G	p.Tyr110*	c.2940delC	p.Pro981Leufs*43	AR	0	1	M	Moderate	Flat	N	N	N	N	NA
	JHLB-8586	Brother	c.330C>G	p.Tyr110*	c.2940delC	p.Pro981Leufs*43		5	7	M	Moderate	Flat	N	Y	N	N	NA
13	JHLB-4166	Proband	c.330C>G	p.Tyr110*	c.3431delG	p.Ala1145Leufs*17	Sporadic	5	15	M	Moderate	Flat	NA	N	N	N	N
14	JHLB-3713	Proband	c.330C>G	p.Tyr110*	c.4073delT	p.Val1358Glyfs*81	Sporadic	5	7	F	Mild	Flat	N	N	N	N	NA
15	JHLB-8287	Proband	c.330C>G	p.Tyr110*	c.4073delT	p.Val1358Glyfs*81	AR	8	8	F	Mild	Flat	N	N	N	N	Y
	JHLB-14540	Brother	c.330C>G	p.Tyr110*	c.4073delT	p.Val1358Glyfs*81		6	6	M	Mild	Flat	NA	NA	NA	NA	NA
16	JHLB-8554	Proband	c.330C>G	p.Tyr110*	c.5219delC	p.His1742Thrfs*127	AR	7	8	M	Mild	Flat	N	Y	N	N	NA
	JHLB-8555	Brother	c.330C>G	p.Tyr110*	c.5219delC	p.His1742Thrfs*127		0	5	M	Moderate	HF gentle	N	Y	N	N	NA
17	JHLB-2064	Proband	c.330C>G	p.Tyr110*	c.5897C>G	p.Ala1966Gly	Sporadic	0	2	M	NA	NA	N	N	N	N	NA
18	JHLB-4682	Proband	c.330C>G	p.Tyr110*	c.6281delC	p.Val2095Cysfs*24	Sporadic	0	13	F	Moderate	Flat	N	NA	N	N	Y
19	JHLB2376	Proband	c.330C>G	p.Tyr110*	c.7021C>T	p.Gln2341*	Sporadic	0	10	M	Moderate	Flat	N	N	N	N	Y
20	HL4496	Proband	c.330C>G	p.Tyr110*	c.7523A>G	p.Asn2508Ser	NA	NA	NA	NA	NA	NA	NA	NA	NA	NA	NA
21	JHLB-7763	Proband	c.330C>G	p.Tyr110*	c.7666C>T	p.Arg2556*	Sporadic	6	12	F	Mild	Flat	N	N	N	N	NA
22	JHLB-6161	Proband	c.330C>G	p.Tyr110*	c.7722C>G	p.Tyr2574*	Sporadic	0	3	F	Moderate	Flat	N	N	N	N	Y
23	JHLB-6369	Proband	c.1196G>T	p.C399F	c.7971+3A>G	.	Sporadic	30	44	M	Moderate	Flat	Y	Y	Y	N	NA
24	JHLB-6063	Proband	c.3431delG	p.A1145Lfs*17	c.4073delT	p.Val1358Glyfs*81	Sporadic	0	3	F	Moderate	HF gentle	N	N	NA	NA	Y
25	JHLB-5379	Proband	c.4073delT	p.V1358Gfs*81	c.4073delT	p.Val1358Glyfs*81	Sporadic	0	0	M	Mild	Flat	N	N	N	N	NA
26	JHLB-4892	Proband	c.4073delT	p.V1358Gfs*81	c.7588C>T	p.Gln2530*	Sporadic	5	11	M	Moderate	Flat	N	N	N	N	NA

All variants are indicated on NM_001277269. AA: amino acid; HL: hearing loss; Y: yes; N: no; NA: not available; *: stop codon.

**Table 3:** Haplotype analysis of the recurrent *OTOG* variant, c.330C>G.

Distance from the c.330C > G Variant (bp)	Allele Frequency in 14KJPN	Marker	Family 2			Family 3	Family 4	Family 6	Family 7
			JHLB- 0441	JHLB- 0442	JHLB- 0443	JHLB- 0580	JHLB- 1308	JHLB- 5053	JHLB- 5086
467019	G:0.63/C:0.37	rs1987694	G/G	G/G	G/G	G/G	G/G	C/G	C/G
393607	G:0.66/A:0.34	rs12576590	A/A	A/A	A/A	A/A	A/A	A/G	A/G
219892	T:0.50/G:0.50	rs11024256	G/T	G/T	G/T	G/T	G/G	G/G	G/G
201848	C:0.51/G:0.49	rs10832775	C/C	C/C	C/C	C/C	C/C	C/C	C/C
177394	C:0.53/G:0.47	rs10832782	C/G	C/G	C/G	C/G	G/G	C/C	C/G
173580	T:0.52/C:0.48	rs9633836	C/T	C/T	C/T	C/T	T/T	C/C	C/T
101954	T:0.65/G:0.34	rs6486370	G/G	G/G	G/G	G/T	G/G	G/G	G/G
86566	C:0.59/T:0.41	rs11024296	C/T	C/T	C/T	C/C	C/C	C/T	C/T
81485	A:0.59/G:0.41	rs2237966	C/T	C/T	C/T	T/T	T/T	C/T	C/T
54803	C:0.73/T:0.27	rs2072233	C/T	C/T	C/T	T/T	T/T	C/T	C/T
15649	A:0.56/G:0.44	rs2237957	T/T	T/T	T/T	T/T	T/T	T/T	T/T
15158	A:0.59/T:0.41	rs4757540	A/A	A/A	A/A	A/A	A/A	A/A	A/A
1338	A:0.58/G:0.42	rs10766410	A/A	A/A	A/A	A/A	A/A	A/A	A/A
79	C:0.56/T:0.44	rs4757543	C/C	C/C	C/C	C/C	C/C	C/C	C/C
0	C:0.996/G:003	*OTOG*:c.330C>G							
37370	A:0.72/G:0.28	rs7116393	A/A	A/A	A/A	A/A	A/A	A/A	A/A
179960	A:0.70/G:0.30	rs7949069	A/G	A/G	A/G	A/G	A/A	A/A	A/G
199806	G:0.63/A:0.37	rs757511	C/T	C/T	C/T	C/T	C/C	C/C	C/T
302936	C:0.69/T:0.31	rs12419230	C/C	C/C	C/C	C/C	C/C	C/C	C/C
329967	A:0.64/G:0.36	rs121704	A/G	A/G	A/G	A/G	A/G	G/G	A/A
373644	C:0.56/T:0.44	rs1468291	C/T	C/T	C/T	C/T	C/T	T/T	C/C
381912	T:0.64/C:0.36	rs1914710	C/T	C/T	C/T	C/T	C/T	C/C	T/T
415228	G:0.56/A:0.44	rs211096	A/G	A/G	A/G	A/G	A/G	A/A	G/G
427221	C:0.64/T:0.36	rs211114	C/T	C/T	C/T	C/T	C/T	T/T	C/C
484625	T:0.54/C:0.46	rs169806	C/T	C/T	C/T	C/T	C/T	C/C	T/T
595928	T:0.51/C:0.49	rs2445164	C/C	C/C	C/C	C/C	C/C	C/T	C/C
603781	A:0.51/G:0.49	rs11024502	G/G	G/G	G/G	A/G	A/G	A/G	G/G
628372	G:0.54/A:0.46	rs1902266	A/A	A/A	A/A	A/G	A/G	A/G	A/A
650836	C:0.57/G:0.43	rs2468803	G/G	G/G	G/G	C/G	C/G	C/G	G/G
711107	T:0.72/A:0.28	rs4638289	T/T	T/T	T/T	T/T	T/T	T/T	T/T

Pink indicates the SNPs with a conserved homozygous status and pale blues indicates heterozygous SNPs or SNPs for which the founder genotype could not be defined.

## Data Availability

The datasets used during the current study are available from the corresponding author upon reasonable request.
